# Developmental Robustness by Obligate Interaction of Class B Floral Homeotic Genes and Proteins

**DOI:** 10.1371/journal.pcbi.1000264

**Published:** 2009-01-16

**Authors:** Thorsten Lenser, Günter Theißen, Peter Dittrich

**Affiliations:** 1 Bio Systems Analysis, Department of Computer Science, Friedrich Schiller University, Jena, Germany; 2 Department of Genetics, Friedrich Schiller University, Jena, Germany; University of Virginia, United States of America

## Abstract

*DEF*-like and *GLO*-like class B floral homeotic genes encode closely related MADS-domain transcription factors that act as developmental switches involved in specifying the identity of petals and stamens during flower development. Class B gene function requires transcriptional upregulation by an autoregulatory loop that depends on obligate heterodimerization of DEF-like and GLO-like proteins. Because switch-like behavior of gene expression can be displayed by single genes already, the functional relevance of this complex circuitry has remained enigmatic. On the basis of a stochastic *in silico* model of class B gene and protein interactions, we suggest that obligate heterodimerization of class B floral homeotic proteins is not simply the result of neutral drift but enhanced the robustness of cell-fate organ identity decisions in the presence of stochastic noise. This finding strongly corroborates the view that the appearance of this regulatory mechanism during angiosperm phylogeny led to a canalization of flower development and evolution.

## Author Summary

The development of organs, their position, and boundaries in multicellular organisms are defined by genes that can sustain their own activation over long periods of time, termed genetic switches. A good case in point is provided by the genetic machinery controlling the development of flowers in higher plants. In *Arabidopsis thaliana* and other plants, a particular class of these genes—*DEF*-like and *GLO*-like floral homeotic genes—regulates the development of petals and stamens. These genes are self-activating via a heterodimer of their protein products, making the activity of each one of them fully bound to the activity of the other one. The reason for their total functional interdependence has long remained unclear, as the expression of both genes is jointly controlled by shared transcription factors in addition to the heterodimer. In principle, one gene alone could provide their switching functionality. In this study, we use computer modeling to show that the obligate heterodimerization mechanism found in *DEF*- and *GLO*-like genes reduces the susceptibility of the genetic switch to failure caused by stochastic noise. This would have provided the system an evolutionary advantage over a single gene with the same functionality.

## Introduction

Depending on the nature of the interactions of their constituents, gene regulatory circuits can display a variety of dynamical behaviors ranging from simple steady states, to switching and multistability, to oscillations. Temporal or spatial patterning during development requires activation of genes at a particular time or position, respectively, and the inhibition in the remaining time or part. Regulatory genes involved in such processes often show a switch-like temporal or spatial dynamics, which requires a direct or indirect positive non-linear feedback of the genes on their own expression, e.g. via dimers of their own product [1]. Switch-like behavior can be displayed by a single gene [2],[3], but many gene regulatory switches have a more complex structure. Due to the small number of molecules involved, these switches are inherently stochastic and their behavior under noisy conditions can strongly depend on their genetic architecture [4]–[6]. In some cases the complex regulatory interactions have been quite well documented, but the functional implications of the corresponding regulatory circuitry have remained enigmatic. A good case in point is provided by some floral homeotic (or organ identity) genes from model plants such as *Arabidopsis thaliana* (thale cress; henceforth termed *Arabidopsis*) and *Antirrhinum majus* (snapdragon; henceforth called *Antirrhinum*).

Floral homeotic genes act as developmental switches involved in specifying organ identity during flower development. According to the ‘ABC model’, three classes of floral organ identity (or homeotic) genes act in a combinatorial way to specify the identity of four types of floral organs, with class A genes specifying sepals in the first floral whorl, A+B petals in the second whorl, B+C stamens (male reproductive organs) in the third whorl, and C alone carpels (female organs) in the fourth floral whorl [7]. The combinatorial genetic interaction of floral homeotic genes may involve the formation of multimeric transcription factor complexes that also include class E (or SEPALLATA) proteins, as outlined by the ‘floral quartet’ model [8].

In *Antirrhinum*, there are two different class B genes termed *DEFICIENS* (*DEF*) and *GLOBOSA* (*GLO*). In *Arabidopsis* these genes are represented by *APETALA3* (*AP3*), the putative orthologue of *DEF*, and *PISTILLATA* (*PI*), the putative *GLO* orthologue. For simplicity, we will refer to *DEF*-like and *GLO*-like genes from here on. *DEF*-like and *GLO*-like genes represent paralogous gene clades that originated by the duplication of a class B gene precursor 200–300 million years ago [9],[10]. All class B genes identified so far, like most other floral homeotic genes, belong to the family of MADS-box genes, encoding MADS-domain transcription factors [11],[12].

Mutant phenotypes reveal that *DEF*-like and *GLO*-like genes are essential for the development of petals and stamens, since *def* and *glo* loss-of-function mutants all produce flowers with petals converted into sepals and stamens transformed into carpels [13]–[17]. When co-expressed in the context of a flower, DEF and GLO are not only required, but even sufficient for specifying petal and stamen identity, as revealed by transgenic studies (e.g., [18]).

**Figure 1:**
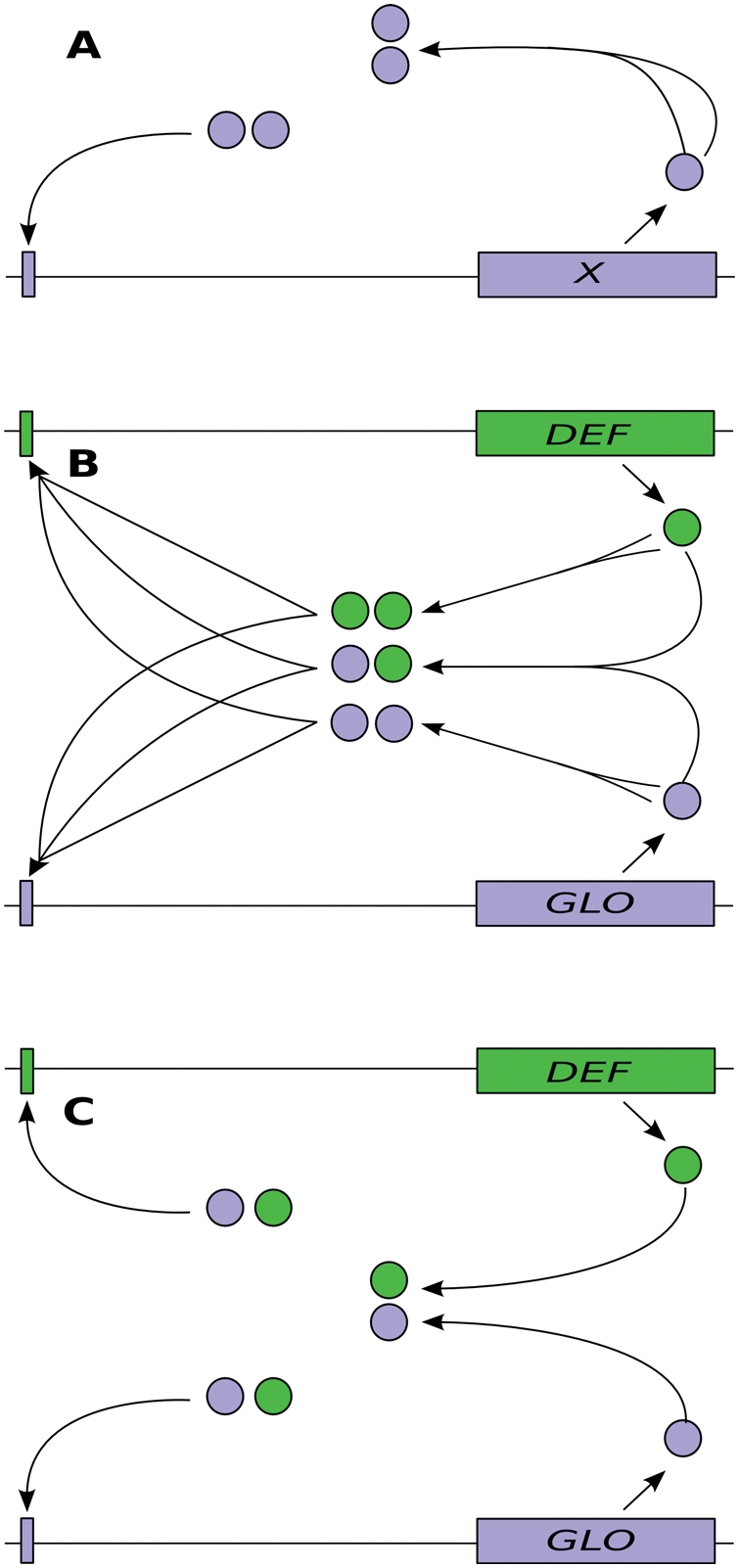
The three types of regulatory mechanisms that are investigated. “DEF” and “GLO” denote DEF-like and GLO-like genes. Large boxes represent the coding regions of genes (neglecting intron-exon structure), small boxes symbolize *cis*-regulatory elements (CArG-boxes). (A) Ancestral state. A single gene *X* is positively regulated by a dimer of its gene product. (B) Intermediate state. After gene duplication, three types of protein dimers can be formed, since both homo- and heterodimerization are possible. All three dimers regulate both genes. (C) Final state. After having lost the homodimerization ability, the remaining heterodimer regulates both genes.

Induction and stable maintenance of switch-gene expression are typically two independent processes, depending on a transient external signal and autoregulation, respectively [19]. Whenever a transient activating signal is above a threshold, the gene activity switches from the OFF- to the ON-state. The signal is required only for initiation, but not for maintenance of gene activity. Due to the autoregulation, the gene's response becomes in a wide range independent of the exact strength of the input signal. During later stages of flower development (in *Arabidopsis* from stage 5 on), mRNA of *DEF*- and *GLO*-like genes is detected only in whorls 2 and 3 [15],[16]. This is so because upregulation and maintenance of class B gene expression in *Arabidopsis* and *Antirrhinum* during later stages of flower development depends on both DEF and GLO, due to an autoregulatory loop involving these proteins (Figure 1C). The proteins encoded by class B genes of *Arabidopsis* and *Antirrhinum* are stable and functional in the cell only as heterodimers, i.e., DEF-GLO complexes, because both nuclear localization and sequence-specific DNA-binding depend on obligate heterodimerization [19],[20]. Class B protein heterodimers bind to specific *cis*-regulatory DNA sequence elements termed ‘CArG-boxes’ (consensus 5′-CC(A/T)_6_GG-3′). Except *PI*, the promoter regions of all class B genes of *Arabidopsis* and *Antirrhinum* contain CArG-boxes that are involved in positively regulating class B gene expression [21]–[23]. These data, together with the total functional interdependence of the two class B gene paralogues, strongly corroborate the hypothesis that positive autoregulatory control of class B genes involves heterodimers of class B proteins that bind to CArG-boxes in the promoters of class B genes (Figure 1C) [14]. Since *PI* lacks CArG-boxes in a minimal promoter region, the autoregulatory feedback may work indirectly in this case [23],[24].

Obligate heterodimerization of their encoded products involved in positive autoregulation explains why *DEF*-like and *GLO*-like genes are functionally non-redundant and totally interdependent. This raises the question as to how and why such a regulatory system originated in evolution. Studies on the interaction of class B protein orthologues from diverse gymnosperms and angiosperms suggested that, following a gene duplication within the class B gene clade, obligate heterodimerization evolved in two steps from homodimerization via facultative heterodimerization [25]. Meanwhile obligate heterodimerization of DEF-like with GLO-like proteins has also been observed outside of the eudicots *Arabidopsis* and *Antirrhinum* in diverse groups of monocots, suggesting that it originated quite early or several times independently during angiosperm evolution [26].

So why then did obligate heterodimerization evolve? In principle, it could represent a neutral change in protein-protein interactions that occurred by random genetic drift [25]. This cannot be excluded at the moment, but for several reasons, it appears not very likely. Even though obligate heterodimerization originated early or several times independently within class B proteins, it did not occur in any other class of floral homeotic proteins, suggesting some kind of functional specificity. Moreover, it occurs within evolutionary especially ‘successful’ (e.g., species-rich) groups of angiosperms, suggesting that it might provide some selective advantage.

Winter et al. [25] suggested that obligate heterodimerization in combination with autoregulation may have provided a selective advantage because of the fixation of class B gene expression patterns and thus the spatial domain of the floral homeotic B-function within the flower during evolution. Mutational changes in the promoter region of only one class B gene that expand the gene's expression domain may leave the late and functionally especially relevant expression domain of the class B genes unchanged, because expression of the other partner would be missing in the ectopic expression domain. Only parallel changes in both types of class B genes, which are much less likely than changes in single genes, could lead to ectopic expression of the B-function under the assumption of obligate heterodimerization and strong autoregulation. Thus obligate heterodimerization may have evolved in parallel, or even as a prerequisite, of the canalization of floral development and thus standardization of floral structure in some groups of flowering plants [25].

Amending this ‘evolutionary’ explanation of obligate heterodimerization, we put forward and test a set of stochastic *in silico* models of class B gene and protein interactions as shown in Figure 1, thus testing the hypothesis that obligate heterodimerization also provides advantages during development by providing robustness against wrong cell-fate decisions caused by stochastic noise. The models enabled us to study the influence of noise in isolation from other factors, and allowed the comparison of three major stages in the envisioned path of evolutionary transitions (Figure 1): (A) One ancestral gene positively regulates its transcription via a homodimer of its own gene product; (B) Two genes positively regulate their transcription via homo- and heterodimers of both types of products; this very likely represents the situation directly after duplication of the ancestral gene; (C) Obligate heterodimerization of the two products for regulation, i.e., the situation in extant *Arabidopsis* and *Antirrhinum*. Since only a small number of individual transcription factors is actually in the nucleus at any time [24],[25], stochastic fluctuations play a large role in the behavior of gene regulatory circuits, and may have an influence on their evolutionary dynamics [5],[27].

Each model consists of a set of reactions for transcription factor binding, transcription, dimerization, and decay (Table S1), where translation is modeled in one step together with dimerization for efficiency (details in Methods section). In turn, each reaction is associated with a propensity function (Tables S2 and S3), which yields the probability of an occurrence of that reaction in a time step. Using the Gillespie algorithm [28], the exact order and timing of reactions is then stochastically determined, based on the propensities. To model transient activation of the circuits, we simulate an inflow of activating molecules (summarizing all different activating transcription factors other than DEF/GLO that act on the respective genes) over 50 minutes of simulated time. After this time, the inflow is switched off and the system equilibrates, i.e., reaches a state in which no change occurs except for stochastic fluctuations (always reached after 72 hours of simulated time). If at this point gene product dimers are still present, the circuit is considered as active (full expression), otherwise it is inactive (no expression of class B genes). Linear stability analysis of the corresponding differential equation system reveals that both the active and the inactive state constitute stable fixed points in all three systems, with an unstable fixed point in between (data not shown).

## Results/Discussion

**Figure 2:**
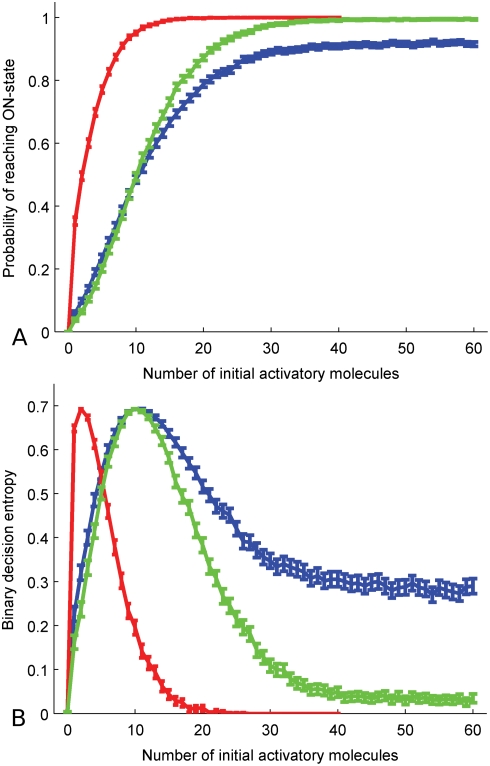
Statistical analysis of 10000 independent runs for each given number of regulatory input molecules. (A) Probability of reaching ON-state and (B) uncertainty of ON-OFF decision for one autoregulatory gene (blue), the two-gene circuit immediately after duplication (red) and with obligate heterodimerization (green). Shown are estimated values and 99% confidence intervals. Parameters are as in Table S2.

The activation of the *DEF* and *GLO* genes depends on a temporally limited concerted action of many more genes and proteins besides the class B genes themselves, which have been described from an evo-devo perspective [12] and by mathematical modeling [29]. To keep the focus on the self-regulation of the genetic switch, we summarize these in one common or two distinct activators for both genes, respectively. In the first experiment we used a common regulator to temporally activate both genes, and investigated the switching behavior of the three circuits with regard to the number of available activatory input molecules. Looking at the probability of reaching full expression (Figure 2A), the most probable state in the one-gene circuit switches from no steady-state expression (resulting in a non-class B cell identity) to full expression (class B, i.e., petal or stamen cell) at approximately 10 input molecules. Gene duplication without further mutational changes leads to a 3 times lower switching threshold (Figure 2A), which may entail a drastically increased zone of class B gene expression in the flower. Mutations leading to obligate heterodimerization again increase the activation threshold to the previous level, thus restoring the class B gene expression region (Figure 2A). Therefore, in contrast to the facultative heterodimerization circuit, obligate heterodimerization results in the same switching threshold and thus the same domain of expression as just one autoregulatory gene. This result is in contradiction to an intuitive expectation that two genes can produce twice as many dimers as a single gene. With obligate heterodimerization, however, the heterodimers assemble from translated products of one DEF and one GLO mRNA intermediate, while the homodimer in the one-gene system is produced from two translated proteins of the same type. Because mRNA is not used up in translation, this leads to equal production rates for the heterodimer in the obligate heterodimerization system and the homodimer in the one-gene system.

To look at the robustness of the switching decision against stochastic noise, we calculated the decision uncertainty (binary entropy), thus more uncertainty implies less robustness. Focusing on the two circuits with identical expression domains, this uncertainty is nearly equal in the first and third circuit for small numbers of activatory input molecules, until the peak of uncertainty is reached. In contrast, the probability for a decision against class B gene mediated cell identity despite large numbers of activatory input molecules is significantly higher in the one-gene circuit than in the circuit with obligate heterodimerization. With 60 activatory molecules, the probability for such a ‘false negative’ in the former circuit is still 10%, while the latter one achieves nearly 100% correct decisions under our conditions (Figure 2B).

Hence, comparing one autoregulatory class B gene with the circuit after duplication and reduction to obligate heterodimerization, our model suggests that an important difference lies in the response to larger numbers of activatory molecules, where the latter system exhibits a clearly reduced tendency to switch off by mistake. This is explained by the fact that although the circuit needs both DEF-like and GLO-like proteins to sustain activation, its two pools of gene products provide a buffer to temporary stochastic failure of one of the two genes. This is especially important during the initial phase of activation, where circuits that are supposed to lock themselves into permanent expression are susceptible to a run of ‘bad luck’, i.e., the supposedly-active genes are inactive over a longer period of time. Obligate heterodimerization of gene products therefore provides a way to gain robustness against wrong cell identity decisions while retaining the original expression domain of one autoregulatory gene.

**Figure 3:**
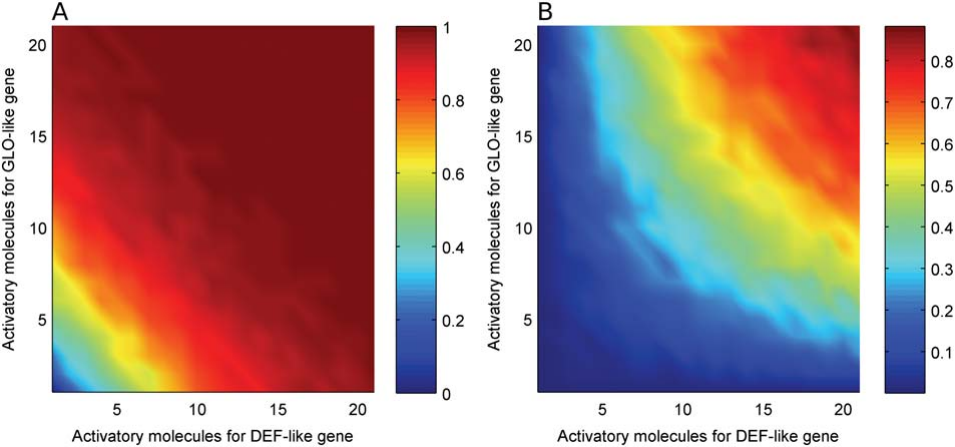
Switching behavior of the two-gene circuits simulating two independent inputs, under (A) facultative heterodimerization, and (B) obligate heterodimerization. X- and Y-axes denote the number of available input molecules for DEF and GLO, respectively. The probability of ending in the ON state is indicated by color: blue is low, red is high. Parameters are as in Table S2.

Even though the mechanisms of the initial activation of *DEF*-like and *GLO*-like genes appear to be quite similar, they are very likely not identical [23], since the initial expression patterns of *DEF*- and *GLO*-like genes are slightly different. In *Arabidopsis* flowers at an early developmental stage 3, *AP3* (*DEF*-like) is expressed in the organ primordia of whorls 2 and 3, but also in parts of whorl 1, while *PI* (*GLO*-like) is expressed in whorls 2–4 at the same stage [15],[16]. In contrast, the *AP3* orthologue *DEF* is expressed weakly in the organ primordia of whorl 4 (carpels) and very weakly in those of whorl 1 (sepals), while the *PI* orthologue *GLO* is expressed in sepal but not carpel primordia of early stages during *Antirrhinum* flower development [14],[19]. To investigate the consequences of independent input into both genes, we explored a model setting in which the *DEF*-like and the *GLO*-like gene are activated independently by two input signals. Our experiments showed that immediately after gene duplication, the mode of integration represents a logical ‘OR’, meaning that both inputs can independently switch on the circuit (Figure 3A). In this case, each input has the role of the one input present before duplication. After the transition to obligate heterodimerization, a logic ‘AND’ function is achieved (Figure 3B), thus both inputs are needed for activation.

In conclusion, we are providing here, to the best of our knowledge, the first rationale, developmental genetic explanation for the intricate design of a genetic switch controlling class B floral homeotic gene expression in core eudicots, involving obligate heterodimerization and positive autoregulatory feedback of two duplicate genes or their protein products, respectively. The increased robustness against unwanted deactivation by chance found in case of obligate heterodimerization strongly suggests that this mechanism has a distinct advantage when the number of available regulatory molecules is small, leading to less cells of wrong identity in a floral organ and therefore to sharper organ identity transitions. It should be noted that since the mathematical model applies to any system with obligate heterodimerization and positive feedback, the conclusions drawn here also transfer to any such system. However, to the best of our knowledge, the phenomenon of obligate heterodimerization together with positive feedback seems quite rare in genetic regulation outside of flower development, potentially due to the high cost of maintaining this system together with a strong dependence of the predicted fitness gain on external factors that might be specific for the situation depicted here.

In the standard ABC model, class A and C genes are mutually antagonistic [7],[30], while class B genes have no floral homeotic ‘repressor’, possibly explaining the class-specific need for sharpened expression domains and thus obligate heterodimerization, which is not found in the other two gene classes. However, Zhao et al recently reported that the antagonistic expression of class A and class C genes is involved in defining the expression domain of class B genes in *Arabidopsis* [31], suggesting that our observation may not be sufficient to explain the obligate heterodimerization of class B proteins. Taking a different perspective, the evolution of a regulatory ‘AND’ function out of an ‘OR’ function may have provided the plant with a more stringent control of the class B floral homeotic genes depending on different induction signals. The fact that there must be different inputs into *DEF*- and *GLO*-like genes is obvious from gene expression studies (see above), but its functional importance may have escaped the attention of previous investigations because of the coordinate upregulation and functional importance of *DEF*- and *GLO*-like genes in the second and third floral whorl. Our results suggest that identifying these different induction pathways, and clarifying their molecular mechanisms (e.g., *trans*-acting factors and *cis*-regulatory DNA motifs in *DEF*-like and *GLO*-like genes being involved) would enable an important step forward in understanding class B floral homeotic gene function in flowering plants.

The functional implication of these different input signals, and hence also of our hypothesis, could be tested by transgenic experiments. For example, *Arabidopsis* class B gene mutants in which both the *AP3* and the *PI* gene have been brought under the control of the *AP3* or the *PI* promoter rather than every gene under its own promoter (as in the wild-type) should affect the spatial or temporal development of petals or stamens, or both. Transgenic plants mutated at the *pi* locus (*pi-1*) in which wild-type PI is expressed under control of the *AP3* promoter (*5D3*) have already been reported [32]. These plants were used only as control for other experiments and have therefore not been described in much detail concerning the traits of interest here. However, it is clear that the *5D3::PI pi-1*/*pi-1* plants do not just show petals in the second floral whorl and stamens in the third floral whorl, as wild-type plants do; rather, they frequently develop sepal/petal mosaics in the second whorl, and mosaic organs or even carpels in the third whorl. These observations support our hypothesis concerning the functional importance of different induction pathways controlling the expression of *DEF*- and *GLO*-like genes for a proper development of organ identity in whorls two and three. More detailed analyses should be done to better understand how exactly the transgenic plants deviate from wild-type plants, and why. In addition, complementary transgenic studies in which AP3 is expressed under control of the *PI* gene promoter (*pPI*) should be performed in order to determine whether the *pPI::AP3 ap3*/*ap3* plants have also developmental defects. The construction of a transgenic plant with switched promoters (i.e., *pAP3::PI pPI::AP3 ap3/ap3 pi/pi*) would also be of great interest. Due to the apparently symmetric roles of *AP3* and *PI*, one might speculate that this phenotype shows less deviation from the wild type than the transgenic plants with both genes under the control of a single promoter.

If the origin of obligate heterodimerization of class B proteins during evolution provided some plants with selective advantages, one may expect that this had an impact on the molecular evolution of these proteins, which indeed seems to be the case. Class B floral homeotic proteins are MIKC-type MADS-domain proteins characterized by a defined domain structure, including a MADS (M), Intervening (I), Keratin-like (K) and a C-terminal (C) domain [11],[12]. The K-domain mediates heterodimerization of GLO- and DEF-like proteins and has been postulated to fold into three amphipatic α-helices termed K1, K2 and K3 [33]. In accordance with the expectations mentioned above, phylogenetic data indicate that after the duplication leading to *DEF*-like and *GLO*-like gene lineages, positive selection acted on the sections of these genes encoding the K-domain [34]. Intriguingly, one site under positive selection [34] is in a subdomain of K1 (“position 97-102” according to ref. [33]) proposed to be critical for heterodimerization specificity of DEF- and GLO-like proteins, as revealed by yeast two-hybrid analyses [33].

Given that the duplicates resulting from one homodimerizing protein would be capable of homo- as well as heterodimerization, our results suggest that positive selection should have enforced the loss of the homodimerization ability, since our model with duplicated class B genes and obligatory heterodimerization implies a sharper switching characteristic and a more constrained domain of class B gene expression than the one with facultative heterodimerization. It has been proposed that within the subdomain of K1 mentioned above, the interaction of Glu-97 in PI and Arg-102 in AP3 facilitates specific heterodimerization between AP3 and PI and prevents formation of homodimers [33]. For these sites, however, positive selection has not been detected [34]. Clearly, the relationships between the molecular evolution and biophysical interactions of DEF- and GLO-like proteins deserve more detailed studies in the future.

All in all, our findings strongly support the view that the unexpected complexity of the floral homeotic gene switch considered here was not simply produced by random genetic drift but evolved because it provided the plant with a clear selective advantage. This might have led to the establishment of this regulatory motif in a whole range of plant species. In line with this notion, it is intriguing that at least some basal angiosperms do not have sharp, but ‘fading borders’ of expression of orthologues of *DEF*-like and *GLO*-like genes as well as gradual transitions in organ identity [35]. This underlines the hypothesis [25] that the mechanism described here improves developmental robustness and thus helped to canalize the development and hence also the evolution of flowers within angiosperm evolution.

## Methods

The model investigated in this work is simulated using the Gillespie algorithm [28], implemented as a C++ function linked to MATLAB (The MathWorks, Inc. 2008). This method, which simulates an exact instance of the stochastic master equation, explicitly accounts for each reaction event and thus represents stochastic effects in full detail. A list of all modelled reactions is given in Table S1, and the full model is shown in Figure S2. Transcription factor binding and unbinding are simple reaction processes, where we assume that exactly one functional copy of both *DEF* and *GLO* genes are available. For simplicity, we assume that only activated DNA is transcribed; however, experiments with basal transcription rates have led to qualitatively similar results. The decision to model translation and dimerization in one step was taken to simplify the model while keeping the focus on transcriptional rather than translational regulation. This entails that we only model *DEF* and *GLO* mRNA and the dimerized proteins, but not the single DEF and GLO proteins. The slight loss of accuracy here has been unavoidable, as we needed to keep the model computationally tractable for the large numbers of replicated experiments. All constituents of the model decay with a linear rate. For details on all kinetic rate constants, see the Text S1 and Tables S1–S3. We conducted 10,000 experiments for each parameter combination.

The different types of regulation are achieved by enabling or disabling the binding and activation of one type of gene by either a transcription factor homodimer produced by itself, a heterodimer of the products of both genes, or a homodimer of the proteins encoded by the other gene. Concerning initial activation, the class B genes are regulated by a number of (possibly interacting) transcription factors, some of which are still unknown. Since the aim of this contribution is to investigate the effect of autoregulation on gene activity, we summarize the effects of all upstream transcription factors in two specific input factors, *I_DEF_* and *I_GLO_,* and a common input factor, *I_C._*

As developmental switches, the B-genes are transiently activated by their inputs, which are switched off after activation. Depending on the level of gene activity reached by that time, this activity either stays high or decays to a low value again, corresponding to on- and off-states of the genes. To model the transient activation, an inflow of (on average) *N* activatory molecules (of type *I_DEF_*, *I_GLO_* or *I_C_*, respectively) over a period of *T* minutes was simulated. After time *T*, the inflow is switched off and the system is left alone, reaching steady state. Figure S1 shows example time courses for all three modes of regulation considered here.

All three systems investigated in this work represent autoactivatory circuits, which are used by the plant to establish the expression (ON-state) or non-expression (OFF-state) of homeotic genes in certain floral whorls. Therefore, a decision has to be made, depending on the number of activatory input molecules initially coming into the system. For low numbers of input molecules, the decision should be ‘OFF’, for higher numbers it should be ‘ON’.

To measure the uncertainty of this decision, we use the binary entropy function. Let *X* be a random variable that takes value 1 with probability *p*, value 0 with probability 1−*p,* i.e., a Bernoulli trial. The entropy of *X* is defined as
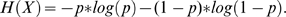
In our case, *X* taking value 1 means that the system reaches ON-state, value 0 means OFF-state. Repeating the simulation 10,000 times, we compute the probability *p* for each specific number *N* of activatory input molecules *I_C_* (Figure 2A). Using the formula above, this translates to the binary entropy, or decision uncertainty (Figure 2B).

Alternative approaches which could potentially lead to additional insights into the functionality of the DEF-GLO system include the application of control theory [36] or an analytical calculation of the first and second stochastic moments, which should confirm the experimental results in this paper.

## Supporting Information

Figure S1 Single runs from all three modes of regulation. Top: one single gene, middle: two genes directly after duplication, bottom: obligate heterodimerization of the transcription factors. Lines in yellow and black show the inputs *I_DEF_ and I_GLO_*, which are switched off after 200 sec.
(2.01 MB TIF)

Figure S2 The full model showing all regulatory parameters. The three different model instances are generated by setting the rate constants to zero or one according to Table S3.
(0.37 MB TIF)

Table S1 A summary of all reactions in the model. Given are the reaction equations and their associated propensity functions. In the one gene model, only gene *DEF* is considered, standing as a surrogate for the ancestral gene of both *DEF* and *GLO*. [X] denotes the number of particles of chemical X in the system. Genes are specified in italics, mRNA in small letters, and proteins in capitals. TF_DEF_ and TF_GLO_ summarize the transcription factors acting on the genes in the specific model, e.g. in the obligatory heterodimerization model, TF_DEF_ = TF_GLO_ = DEF-GLO, while in the system after duplication TF_DEF_ = TF_GLO_ = {DEF-DEF, GLO-GLO, DEF-GLO}.
(0.11 MB DOC)

Table S2 Parameters that are kept constant in all experiments. β is the production propensity for gene products for both genes when they are activated, while β_0_ is their base-level production rate. kon and koff give the binding and unbinding propensities of regulatory dimers to both genes, while d is the decay rate uniformly used for mRNA, dimers and initial activatory molecules.
(0.11 MB DOC)

Table S3 Parameters that are varied between the three experiments. a_ij_ and b_ij_ are binary parameters that determine which types of dimers regulate which gene, while k_ij_s describe the stochastic rate constants in the dimerization propensities for all combinations of monomers.
(0.11 MB DOC)

Text S1 Details on kinetic parameters.
(0.05 MB DOC)
