# Supplementary figures and images for: Developmental Robustness by Obligate Interaction of Class B Floral Homeotic Genes and Proteins
Source: PLoS Comput Biol. 2009 Jan 16;5(1):e1000264. doi: 10.1371/journal.pcbi.1000264 (PMC2612583; doi:10.1371/journal.pcbi.1000264)

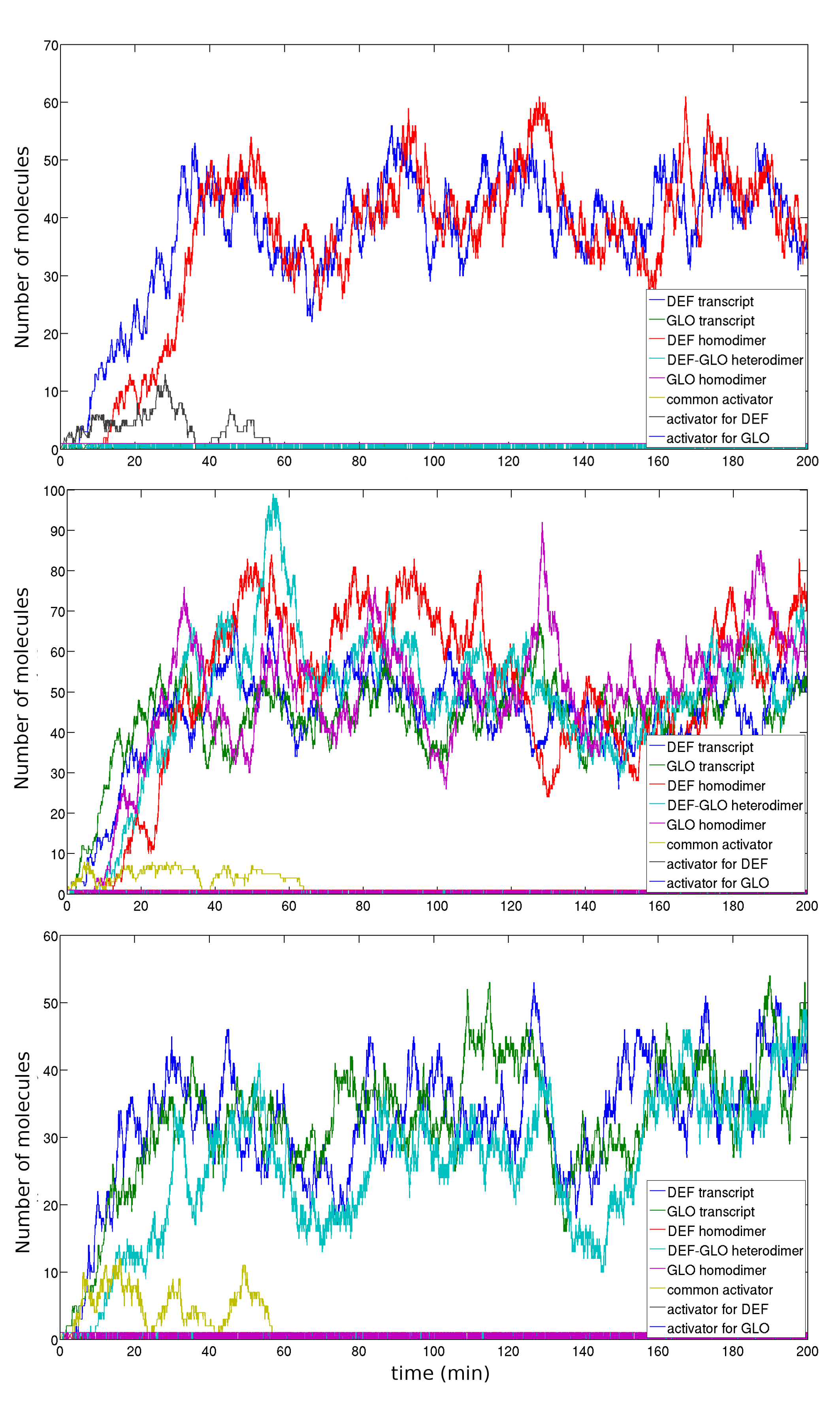

Supplement: Figure S1 — Single runs from all three modes of regulation. Top: one single gene, middle: two genes directly after duplication, bottom: obligate heterodimerization of the transcription factors. Lines in yellow and black show the inputs IDEF and IGLO, which are switched off after 200 sec. (2.01 MB TIF) [file pcbi.1000264.s001.tif]

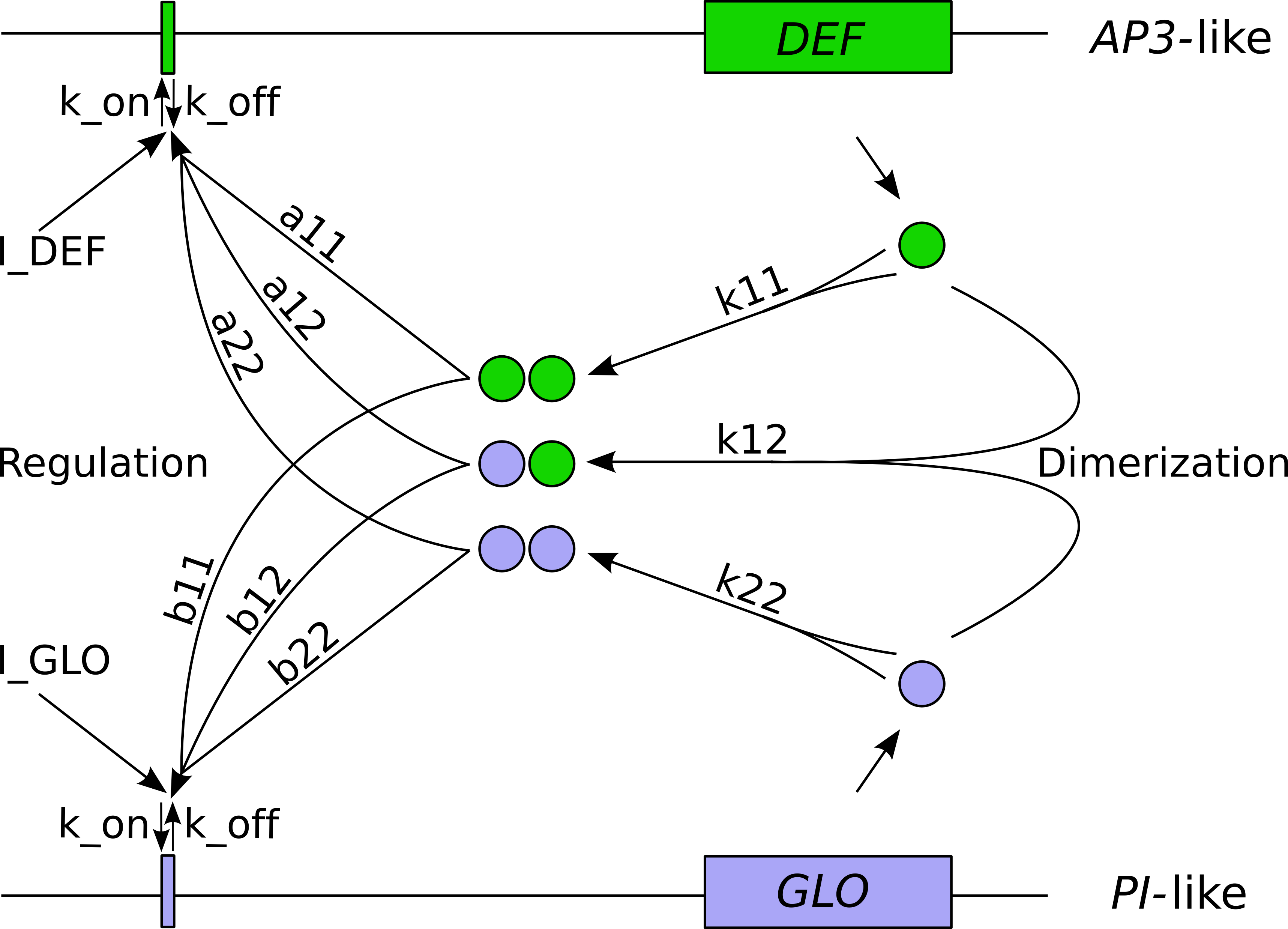

Supplement: Figure S2 — The full model showing all regulatory parameters. The three different model instances are generated by setting the rate constants to zero or one according to Table S3. (0.37 MB TIF) [file pcbi.1000264.s002.tif]
