# Supplementary material for: Developmental Robustness by Obligate Interaction of Class B Floral Homeotic Genes and Proteins
Source: PLoS Comput Biol. 2009 Jan 16;5(1):e1000264. doi: 10.1371/journal.pcbi.1000264 (PMC2612583; doi:10.1371/journal.pcbi.1000264)
Supplement: Table S1 — A summary of all reactions in the model. Given are the reaction equations and their associated propensity functions. In the one gene model, only gene DEF is considered, standing as a surrogate for the ancestral gene of both DEF and GLO. [X] denotes the number of particles of chemical X in the system. Genes are specified in italics, mRNA in small letters, and proteins in capitals. TFDEF and TFGLO summarize the transcription factors acting on the genes in the specific model, e.g. in the obligatory heterodimerization model, TFDEF = TFGLO = DEF-GLO, while in the system after duplication TFDEF = TFGLO = {DEF-DEF, GLO-GLO, DEF-GLO}. (0.11 MB DOC) [file pcbi.1000264.s003.doc]

**Table S1.** A summary of all reactions in the model. Given are the reaction equations and their associated propensity functions. In the one gene model, only gene *DEF* is considered, standing as a surrogate for the ancestral gene of both *DEF* and *GLO*. [X] denotes the number of particles of chemical X in the system. Genes are specified in italics, mRNA in small letters, and proteins in capitals. TF_DEF_ and TF_GLO_ summarize the transcription factors acting on the genes in the specific model, e.g. in the obligatory heterodimerization model, TF_DEF_ = TF_GLO_ = DEF-GLO, while in the system after duplication TF_DEF_ = {DEF-DEF, GLO-GLO, DEF-GLO}.

| Process | Reaction | Propensity |
| --- | --- | --- |
| TF-binding | *DEF* + TF_DEF_ -> *DEF**  *GLO* + TF_GLO_ -> *GLO** | k_on_ * [TF_DEF_]  k_on_ * [TF_GLO_] |
| TF-unbinding | *DEF** -> *DEF* + TF_DEF_  *GLO** -> *GLO* + TF_GLO_ | k_off_  k_off_ |
| Transcription | *DEF** -> *DEF** + def  *GLO** -> *GLO** + glo | β  β |
| Translation + Dimerization | def + def -> def + def + DEF-DEF  glo + glo -> glo + glo + GLO-GLO  def + glo -> def + glo + DEF-GLO | k_11_ * [def] * ([def]-1)  k_22_ * [glo] * ([glo]-1)  k_12_ * [def] * [glo] |
| Decay | def -> 0  glo -> 0  DEF-DEF -> 0  GLO-GLO -> 0  DEF-GLO -> 0 | d * [def]  d * [glo]  d * [DEF-DEF]  d * [GLO-GLO]  d * [DEF-GLO] |
