# Supplementary material for: Developmental Robustness by Obligate Interaction of Class B Floral Homeotic Genes and Proteins
Source: PLoS Comput Biol. 2009 Jan 16;5(1):e1000264. doi: 10.1371/journal.pcbi.1000264 (PMC2612583; doi:10.1371/journal.pcbi.1000264)
Supplement: Table S2 — Parameters that are kept constant in all experiments. β is the production propensity for gene products for both genes when they are activated, while β0 is their base-level production rate. kon and koff give the binding and unbinding propensities of regulatory dimers to both genes, while d is the decay rate uniformly used for mRNA, dimers and initial activatory molecules. (0.11 MB DOC) [file pcbi.1000264.s004.doc]

**Table S2.** Parameters that are kept constant in all experiments. β is the production propensity for gene products for both genes when they are activated, while β_0_ is their base-level production rate. k_on_ and k_off_ give the binding and unbinding propensities of regulatory dimers to both genes, while d is the decay rate uniformly used for mRNA, dimers and initial activatory molecules.

| Parameter (Unit) | Value |
| --- | --- |
| β (molecules/min) | 10.0 |
| β_0_ (molecules/min) | 0 |
| k_on_ (min^-1) | 0.8 |
| k_off_ (molecules/min) | 8 |
| d (min^-1) | 0.2 |
