# Supplementary material for: Developmental Robustness by Obligate Interaction of Class B Floral Homeotic Genes and Proteins
Source: PLoS Comput Biol. 2009 Jan 16;5(1):e1000264. doi: 10.1371/journal.pcbi.1000264 (PMC2612583; doi:10.1371/journal.pcbi.1000264)
Supplement: Table S3 — Parameters that are varied between the three experiments. aij and bij are binary parameters that determine which types of dimers regulate which gene, while kijs describe the stochastic rate constants in the dimerization propensities for all combinations of monomers. (0.11 MB DOC) [file pcbi.1000264.s005.doc]

**Table S3.** Parameters that are varied between the three experiments. a_ij_ and b_ij_ are binary parameters that determine which types of dimers regulate which gene, while k_ij_s describe the stochastic rate constants in the dimerization propensities for all combinations of monomers.

| Parameter (Unit) | One Gene | After Duplication | Obligatory Heterodimerization |
| --- | --- | --- | --- |
| a_11_ (proportion) | 1.0 | 1.0 | 0 |
| a_12_ (proportion) | 0 | 1.0 | 1.0 |
| a_22_ (proportion) | 0 | 1.0 | 0 |
| b_11_ (proportion) | 0 | 1.0 | 0 |
| b_12_ (proportion) | 0 | 1.0 | 1.0 |
| b_22_ (proportion) | 0 | 1.0 | 0 |
| k_11_ (min*l)^-1 | 0.01 | 0.01 | 0 |
| k_12_ (min*l)^-1 | 0 | 0.01 | 0.01 |
| k_22_ (min*l)^-1 | 0 | 0.01 | 0 |
