# Supplementary material for: Developmental Robustness by Obligate Interaction of Class B Floral Homeotic Genes and Proteins
Source: PLoS Comput Biol. 2009 Jan 16;5(1):e1000264. doi: 10.1371/journal.pcbi.1000264 (PMC2612583; doi:10.1371/journal.pcbi.1000264)
Supplement: Text S1 — Details on kinetic parameters. (0.05 MB DOC) [file pcbi.1000264.s006.doc]

# Supporting Information

# Developmental robustness by obligate interaction of class B floral homeotic genes and proteins

**Thorsten Lenser,^1*^ Günter Theißen,^2^ Peter Dittrich^1^**

^1^Bio Systems Analysis, Institute of Computer Science, Friedrich Schiller University,
D-07743 Jena, Germany

^2^Department of Genetics, Friedrich Schiller University, D-07743 Jena, Germany

^*^To whom correspondence should be addressed. E-mail: thlenser@inf.uni-jena.de

**This PDF includes:**

Text: Details on kinetic parameters

Supplemental references

### Supporting text: Details on kinetic parameters

It is important to note that since we are using a stochastic framework, the rate parameters given in Table S2 and S3 are *stochastic* rate constants rather than deterministic ones. As already shown by Gillespie [1], a consequence of this is that the rate constants for first-order reactions (transcription, decay) are given in “1/min on average”, but are otherwise equivalent to deterministic rates, whereas the rate constants for dimerization (second-order) are given in “1/(min * l) on average”, since the volume in which the reaction takes place (given in l) plays a role.

All except the interaction parameters describe stochastic rates, i.e. they characterize the average rate of the corresponding reactions. In other words, if reaction *R: M1 + M2 -> 2*M1* has rate constant *c*, then *c*dt* is the probability that a specific pair of molecules of types M1 and M2 will react in the short time-step *dt*. Parameters *k_ij_* summarize the processes of transport out of the nucleus, translation, dimerization, and transport back into the nucleus into one number, which yields a process occurring in the time-scale of one hour (with given parameters, it takes ~69 min to transcribe 50% of a given amount of mRNA in the nucleus to gene products, dimerize outside of the nucleus, and move back in).

Most important for the behaviour of the model are the transcription factor binding and unbinding rates *k_on_*  and *k_off_.* Their ratio K_D_ = *k_off_ / k_on_*, often called the “dissociation constant”, characterizes the approximate threshold between ON-state and OFF-state of the whole system. This can be seen by noting that in a bimolecular binding reaction

A + B ↔ AB,

the dissociation constant K_D_ denotes the equilibrium ratio between the concentrations of unbound A and B and the dimer AB:

K_D_ = [A]x[B] / [AB]

(where [X] denotes the concentration of chemical X). In our case, A is the gene (of which only one copy exists), B is the transcription factor, and AB the activated form of the gene. Since we are dealing with discrete particle numbers, [AB] denotes the probability that the gene is activated by the transcription factor. Also note that [A] + [AB] = 1 holds. Combining all this yields

K_D_ = (1-[AB])x[B] / [AB]

and thus

[AB] = [B] / (K_D_ + [B]).

Since [AB] is the activation probability of the gene, we conclude that K_D_ is exactly the concentration of the transcription factor that is needed to activate the gene with probability 0.5, and therefore the threshold for activation.

When the ratio of *k_off_* and *k_on_* is kept constant, their absolute values strongly influence the amount of randomness in the system. The smaller their absolute values are, the rarer binding/unbinding events become, which gives these rare events a large influence on system behaviour. On the other hand, when binding and unbinding are fast, these processes can be seen as in equilibrium, reducing their stochastic influence.

In contrast to the chemical parameters above, the interaction parameters *a_ij_* and *b_ij_* are purely phenomenological. They describe the proportion of dimers that are involved in the regulation of the two transcription sites, according to the full model shown in Fig. S2. In this work, they are limited to the states “dimer can/cannot regulate site”, i.e., they are set to either one or zero. Parameter *a_11_* represents the influence of the DEF-DEF homodimer, *a_12_* the influence of the DEF-GLO heterodimer, and *a_22_* the one of the GLO-GLO homodimer, all on the *DEF*-like gene. In an analogous fashion, *b_ij_*s denote the influences on the *GLO*-like gene. For example, if *a_12_=1*, the DEF-GLO heterodimer can bind and activate the *DEF*-like gene, while this binding and activation is disabled for *a_12_=0*.

Even though the model gives a quantitative description of the reaction processes, the results can only be taken qualitatively since the exact kinetic parameters have not yet been measured in plants. The parameters used in this study have been chosen in accordance with experimental evidence from other systems [2-5], especially the overview table 2.1 from (4). Our parameter choices ensure that the model replicates the time-scales involved in homeotic gene regulation, i.e. the main processes involved happen on a timescale of hours. We also varied the parameters in a range of 10% up or down and did not observe a significant change in the model behavior.

### Supplemental references

1. Gillespie DT (1977) Exact Stochastic Simulation of Coupled Chemical Reactions. J Phys Chem 81:2340-2361.

2. Alon U (2006) An Introduction to Systems Biology: Design Principles of Biological Circuits. Chapman & Hall/CRC.

3. Kaern M, Elston TC, Blake WJ, Collins JJ (2005) Stochasticity in gene expression: from theories to phenotypes. Nat Rev Genet 6:451-464.

4. Smolen P, Baxter DA, Byrne JH (1998) Frequency selectivity, multistability, and oscillations emerge from models of genetic regulatory systems. Am J Physiol 274:C531-42.

5. Tian T, Burrage K (2006) Stochastic models for regulatory networks of the genetic toggle switch. Proc Nat Acad Sci USA 103:8372-8377.
